# Predictors of surgical site infection after pancreaticoduodenectomy

**DOI:** 10.1186/s12876-020-01350-8

**Published:** 2020-06-26

**Authors:** Wikran Suragul, Narongsak Rungsakulkij, Watoo Vassanasiri, Pongsatorn Tangtawee, Paramin Muangkaew, Somkit Mingphruedhi, Suraida Aeesoa

**Affiliations:** Department of Surgery, Faculty of Medicine, Ramathibodi Hospital, Mahidol University, 270 Rama VI Road, Ratchathewi, Bangkok, 10400 Thailand

**Keywords:** Pancreaticoduodenectomy, Risk factors, Surgical wound infection

## Abstract

**Background:**

Surgical site infection (SSI) is one of the most common complications after pancreaticoduodenectomy (PD). Thus, it is beneficial to preoperatively identify patients at high risk of developing SSI. The primary aim of the present study was to identify the factors associated with SSI after PD, and the secondary aim was to identify the adverse outcomes associated with the occurrence of SSI.

**Methods:**

A single-centre retrospective study was conducted. All 280 patients who underwent PD at our institution from January 2008 to December 2018 were enrolled. Demographic and perioperative data were reviewed, and the potential risk factors for developing SSI and the adverse outcomes related to SSI were analysed.

**Results:**

A total of 90 patients (32%) developed SSI. Fifty-one patients developed incisional SSI, and 39 developed organ/space SSI. Multivariate logistic analysis revealed that the significant risk factors for developing incisional SSI were preoperative biliary drainage (odds ratio, 3.04; 95% confidence interval, 1.36–6.79; *p* < 0.05) and postoperative pancreatic fistula (odds ratio, 2.78; 95% confidence interval, 1.43–5.38; *p* < 0.05), and the risk factors for developing organ/space SSI were preoperative cholangitis (odds ratio, 10.07; 95% confidence interval, 2.31–49.75; *p* < 0.05) and pancreatic fistula (odds ratio, 6.531; 95% confidence interval, 2.30–18.51; *p* < 0.05). *Enterococcus* spp*.*, *Escherichia coli* and *Klebsiella pneumoniae* were the common bacterial pathogens that caused preoperative cholangitis as well as SSI after PD. The patients in the SSI group had a longer hospital stay and a higher rate of delayed gastric emptying than patients in the non-SSI group.

**Conclusions:**

The presence of postoperative pancreatic fistula was a significant risk factor for both incisional and organ/space SSI. Any efforts to reduce postoperative pancreatic fistula would decrease the incidence of incisional SSI as well as organ/space SSI after pancreaticoduodenectomy. Preoperative biliary drainage should be performed in selected patients to reduce the incidence of incisional SSI. Minimizing the occurrence of preoperative cholangitis would decrease the incidence of developing organ/space SSI.

## Background

Pancreaticoduodenectomy (PD) is a standard operation for the treatment of periampullary cancer and some benign diseases. Despite the substantial improvement in mortality related to this operation, the morbidity is still as high as nearly 50% [1–3]. One of the most common complications after PD is surgical site infection (SSI). SSI prolongs the length of hospital stay, increases costs [4], and may also influence overall survival [5]. Therefore, it is crucial to understand the factors that contribute to SSI after PD.

The Guidelines for Prevention of SSI (1999) published by the Centers for Disease Control define SSI as an infection that occurs within 30 or 90 days postoperatively (depending on the type of surgery) that involves the skin or subcutaneous tissue of the incisional region, deep soft tissues (e.g., fascial and muscle layers), or any part of the anatomy (e.g., organs or spaces) other than the incision that was opened or manipulated intraoperatively [6].

The factors identified in previous studies as risk factors for SSI after PD include preoperative biliary drainage [2, 7], pancreatic fistula [8], a high body mass index (BMI) [8], diabetes mellitus [7], blood transfusion [9], malnutrition, and a low serum albumin level [10–12]. As previous studies have revealed varying results, the primary aim of the present study was to identify the factors associated with SSI after PD, and the secondary aim was to identify adverse outcomes related to SSI after PD.

## Methods

### Patients and data collection

A single-centre retrospective study was conducted. The data from 280 patients who underwent PD at Ramathibodi Hospital from 2010 to 2018 were retrospectively reviewed. The demographic and clinical data collected included age, sex, comorbidity, BMI, American Society of Anesthesiology classification, history of smoking, preoperative biliary drainage, preoperative laboratory test results, duration of surgery, blood loss, perioperative complications, history of preoperative cholangitis, length of hospital stay and final pathologic diagnosis. Postoperative pancreatic fistula (POPF) was defined in accordance with the guidelines of the International Study Group of Pancreatic Fistula [13]. .Bile leakage was defined in accordance with the guidelines of the International Study Group of Liver Surgery [14]. The wounds and drains of all patients were routinely examined for signs of infection; when SSI was suspected, additional bacterial cultures were performed, and bacteriologic data were collected. SSI was defined in accordance with the guidelines for the prevention of SSI of the Centers for Disease Control [6]. Patients were further categorized into those with incisional SSI and those with organ/space SSI. The incisional SSI group included those with infections of the skin, subcutaneous tissue, fascia, and muscle. The present study was approved by the Institutional Review Board of Ramathibodi Hospital.

### Perioperative management

Preoperative biliary drainage (PBD) was carried out in patients with clinical cholangitis, jaundice with malnutrition, or a waiting time of longer than 2 weeks; endoscopic biliary drainage (EBD) was used in most cases, while some patients underwent percutaneous transhepatic biliary drainage (PTBD). Bile juice was collected at the time PBD was performed to isolate the causative bacterium in all patients who developed clinical cholangitis, and antibiotics were prescribed based on the bacterial culture results. The biliary stents or percutaneous catheters were changed in the patients who developed cholangitis after PBD. Oral nutritional supplements were given to the malnourished patients. All patients washed their body and hair with chlorhexidine soap (4% chlorhexidine gluconate) in the evening on the day before the operation and in the morning on the operative day. Hair removal was completed just before moving the patient to the operating room. The surgical field was scrubbed with 4% chlorhexidine gluconate and painted with 2% chlorhexidine gluconate in alcohol or 10% povidone-iodine before commencing the operations. Cefoxitin was used as a preoperative prophylactic antibiotic in all cases and was continued for 24 h postoperatively, in accordance with the protocol of Ramathibodi Hospital.

The surgical procedure comprised either pylorus-preserving PD or standard PD. The pancreaticojejunostomy anastomoses were created using both the duct-to-mucosa and the invagination techniques. Pancreatic duct stents were used in all cases, and the size of the stent was adjusted based on the diameter of the pancreatic duct. Prophylactic drains were routinely placed anterior to the pancreaticojejunostomy anastomosis and posterior to the hepaticojejunostomy anastomosis. Two types of drains, a tube drain and a closed-suction drain, were commonly used. However, the number and type of drains depended on surgeon preference. The peritoneal cavity and the subcutaneous tissue were irrigated with 2 l of normal saline before skin closure.

During the postoperative period, the surgical wounds and the clinical status were routinely examined. The drain amylase level was routinely checked on postoperative days 1, 3, and 5, and the drains were removed on postoperative day 5 if there was no pancreatic leakage. In the patients who developed pancreatic fistula, the drains were retained until pancreatic leakage ceased. The patients who developed SSI after surgery received antimicrobial therapy following the results of bacterial cultures of the intraoperative bile juice or the drainage fluid obtained from the infection area. Diet was resumed, depending on the return of bowel function and patient tolerance. There were no major changes in the clinical practices over these 9 years. However, over the last 3 years, the patients were allowed to resume diet earlier than in previous years; sipping water was allowed on postoperative day 1, a liquid diet was allowed on postoperative day 2, and a soft diet was started on postoperative day 3 or day 4 if tolerable by the patient. The modified Blumgart anastomosis technique for the pancreaticojejunostomy anastomosis was the most common technique used over the last 4 years compared to the previous years, in which the most common was the Cattell-Warren technique.

### Statistical analysis

Categorical variables were analysed using Pearson’s χ^2^ test (or Fisher’s exact test if appropriate), while numerical variables were analysed using independent Student’s t-test (or the Mann-Whitney U test if appropriate). Risk factors with a *p* value of < 0.1 in univariate analysis were entered into multivariable analysis with applied logistic regression. Odds ratios (ORs) and 95% confidence intervals (CIs) were calculated to assess the strength of the associations between the variables and the outcomes. The performance of the final model was described using receiver operating characteristic curves. A *p* value of < 0.05 was considered statistically significant. Analyses were performed using the STATA program, version 14 (StataCorp, College Station, TX, USA).

## Results

### Patient characteristics and perioperative outcomes

A total of 280 patients underwent PD during the study period. Table 1 shows the demographic and perioperative data. SSI occurred in 90 of 280 patients (32%), comprising 51 patients (18%) with incisional SSI and 39 (14%) with organ/space SSI. POPF occurred in 143 patients (51%), comprising biochemical leakage in 73 (26%), grade B in 50 (18%), and grade C in 20 (7%). Among the 143 patients with POPF, 67 (46%) developed SSI, and half of them developed organ/space SSI. POPF was significantly more common in the SSI group than in the non-SSI group. Preoperative biliary drainage was performed in 192 of 280 patients (67%), with endoscopic retrograde cholangiopancreatography accounting for 62% of the drainage procedures. Preoperative cholangitis occurred in 18 patients (6.43%); fourteen of the cases were due to biliary stent occlusion, 2 of the cases were due to obstructive jaundice, and 12 out of 18 patients (68%) developed SSI after PD. The number of patients who underwent preoperative biliary intervention was significantly higher in the SSI group than in the non-SSI group. The incidence of delayed gastric emptying was significantly higher in the SSI group than in the non-SSI group, and the BMI of the patients was significantly higher in the SSI group. There was no difference in age, sex, co-morbidities, ASA physical status, preoperative serum blood urea nitrogen levels, serum creatinine levels, serum albumin levels, serum total bilirubin levels, operative time, intraoperative blood loss, post-operative chyle leakage or postoperative bile leakage between the SSI group and the non-SSI group. The median duration of drain placement in the SSI group was significantly longer than that in the non-SSI group (23 days vs 11 days). The median length of hospital stay was significantly longer in the SSI group than in the non-SSI group (28 days vs 14 days).

**Table 1:** Patient characteristics and demographic data

SSI	Total (***n*** = 280)	Non-SSI (***n*** = 190)	SSI (***n*** = 90)	***p*** value
Age in years, mean ± SD	59.98 ± 10.79	59.81 ± 10.70	60.36 ± 11.01	0.687
BMI, median (range)	22.36 (15.11, 38.95)	21.94 (15.11, 38.95)	22.60 (16.22, 34.06)	0.040
Sex, n (%)				
Male	148 (52.86)	100 (52.63)	48 (53.33)	0.913
Female	132 (47.14)	90 (47.37)	42 (46.67)	
Comorbidities, n (%)				
Diabetes mellitus	81 (28.93)	62 (32.63)	19 (21.11)	0.047
Hypertension	108 (38.57)	70 (36.84)	38 (42.22)	0.388
Dyslipidemia	76 (27.14)	56 (29.47)	20 (22.22)	0.203
Renal insufficiency	7 (2.50)	4 (2.11)	3 (3.33)	0.539
ASA class, n (%), *n* = 275				
I	6 (2.18)	3 (1.62)	3 (3.33)	0.811
II	104 (37.82)	69 (37.30)	35 (38.89)	
III	156 (56.73)	106 (57.30)	50 (55.56)	
IV	8 (2.91)	6 (3.24)	2 (2.22)	
V(E)	1 (0.36)	1 (0.54)	0	
Smoking, n (%)	84 (30.00)	56 (29.47)	28 (31.11)	0.780
Previous biliary intervention, n (%)	192 (68.57)	119 (62.63)	73 (81.11)	0.002
WBC, mean ± SD	7838 ± 2670	7953 ± 2665	7605 ± 2680	0.321
Platelet × 10^3^, median (range)	300 (96, 715)	302 (96, 715)	124 (140, 548)	0.068
Hb in g/dL, median (range)	12 (3, 30.2)	12 (3, 30.2)	2.2 (8.5, 23.3)	0.349
BUN in mg/dL, median (range)	13 (4, 57)	13 (4, 57)	7 (5, 29)	0.712
Creatinine in mg/dL, median (range)	0.8 (0.3, 12)	0.8 (0.3, 11)	0.26 (0.33, 12)	0.734
Total bilirubin in mg/d/L, median (range)	1.6 (0.1, 35)	1.75 (0.2, 35)	3.4 (0.1, 30.3)	0.341
Albumin in g/L, mean ± SD	34.35 ± 5.32	34.55 ± 5.65	33.94 ± 4.57	0.377
Pre-operative cholangitis, n(%)	18 (6.43)	6 (3.16)	12 (13.33)	0.001
Operative time in min, median (range)	475 (240, 900)	435 (240, 900)	480 (240, 840)	0.078
Blood loss in ml, median (range)	800 (100, 13,000)	800 (100, 8000)	800 (150, 13,000)	0.757
Complications, n (%)				
Pancreatic fistula				
No	137 (48.93)	114 (60.00)	23 (25.56)	0.000
Yes	143 (51.07)	76 (40.00)	67 (74.44)	
Bile leakage	20 (7.14)	10 (5.26)	10 (11.11)	0.076
Delayed gastric emptying	29 (10.36)	14 (7.37)	15 (16.67)	0.017
Chyle leakage	13 (4.64)	6 (3.16)	7 (7.78)	0.125
Duration of drain placement (day), median (rang) *n* = 238	13 (2, 266)	10 (2, 94)	23 (2, 266)	0.000
LOH in days, median (range)	16 (5, 320)	14 (5, 87)	28 (8, 320)	0.000
Diagnosis, n (%)				
Benign	73 (26.07)	50 (26.32)	23 (25.56)	0.892
Malignant	207 (73.93)	140 (73.68)	67 (74.44)	

*SSI* Surgical site infection, *SD* Standard deviation, *BMI* Body mass index, *WBC* White blood cell count, *Hb* Hemoglobin, *BUN* Blood urea nitrogen, *LOH* Length of hospital stay

### Factors related to surgical site infection after pancreaticoduodenectomy

The patients in the SSI group were divided into 2 groups depending on the depth of SSI: the incisional and the organ/space SSI groups. The independent risk factors for incisional SSI after PD were preoperative biliary drainage (OR 3.04) and POPF (OR 2.78). For the organ/space POPF SSI, POPF (OR 6.53) and preoperative cholangitis (OR 10.72) were the independent risk factors. The results of uni- and multivariate analyses of the factors related to incisional and organ/space SSI are shown in Table 2 and Table 3, respectively.

**Table 2:** Variables associated with **incisional surgical site infection (SSI)**

SSI	Univariate		Multivariate	
	OR (95% CI)	***p*** value	OR (95% CI)	***p*** value
Age in years, mean ± SD	1.006 (0.97–1.03)	0.657		
BMI, median (range)	1.015 (0.94–1.09)	0.677		
Sex, n (%)				
Male	1			
Female	0.751 (0.41–1.37)	0.352		
Comorbidities, n (%)				
Diabetes mellitus	0.737 (0.37–1.43)	0.368		
Hypertension	1.246 (0.68–2.27)	0.473		
Dyslipidemia	0.638 (0.31–1.29)	0.214		
Renal insufficiency	1.690 (0.30–9.47)	0.550		
ASA class, n (%), *n* = 242				
II	1			
III	0.833 (0.45–1.52)	0.554		
Smoking, n (%)	1.504 (0.81–2.78)	0.195		
Previous biliary intervention, n (%)	3.182 (1.47–6.87)	0.003	3.041 (1.36–6.79)	0.007
WBC, mean ± SD	0.958 (0.85–1.08)	0.496		
Platelet ×10^3^, median (range)	0.794 (0.58–1.07)	0.130		
Hemoglobin in g/dL, median (range)	1.058 (0.94–1.17)	0.303		
BUN in mg/dL, median (range)	0.999 (0.95–1.05)	0.971		
Creatinine in mg/dL, median (range)	1.082 (0.84–1.38)	0.532		
Total bilirubin in mg/d/L, median (range)	0.971 (0.92–1.02)	0.260		
Albumin in g/L, mean ± SD	0.999 (0.94–1.05)	0.975		
Pre-operative cholangitis, n(%)	3.375 (0.20–54.82)	0.392	2.647 (0.75–9.23)	0.127
Operative time in min, median (range)	1.205 (0.96–1.50)	0.098		
Blood loss in ml, median (range)	0.980 (0.74–1.28)	0.888		
Complications, n (%)				
Pancreatic fistula				
No	1		1	
Yes	3.250 (1.73–6.09)	0.000	2.783 (1.43–5.38)	0.002
Bile leakage	2.117 (0.73–6.10)	0.165		
Delayed gastric emptying	3.006 (1.28–7.06)	0.012		
Chyle leakage	2.586 (0.84–7.93)	0.097		
Diagnosis, n (%)				
Benign	1			
Malignant	1.040 (0.58–1.84)	0.892		

*SSI* Surgical site infection, *OR* Odds ratio, *CI* Confidence interval, *BMI* Body mass index, *ASA* American Society of Anesthesiologists, *WBC* White blood cell count, *BUN* Blood urea nitrogen

**Table 3:** Variable associated with organ/space surgical site infection (SSI)

SSI	Univariate		Multivariate	
	OR (95% CI)	***p*** value	OR (95% CI)	***p*** value
Age in years, mean ± SD	1.002 (0.96–1.03)	0.891		
BMI, median (range)	1.104 (1.01–1.20)	0.024	1.107 (0.99–1.22)	0.057
Sex, n (%)				
Male	1			
Female	1.507 (0.71–3.18)	0.281		
Comorbidities, n (%)				
Diabetes mellitus	0.284 (0.09–0.84)	0.024		
Hypertension	1.263 (0.59–2.67)	0.542		
Dyslipidemia	0.765 (0.32–1.80)	0.541		
Renal insufficiency	1.453 (0.15–13.42)	0.742		
ASA class, n (%), n = 242				
I	1			
II	0.144 (0.02–0.81)	0.029		
III	0.169 (0.03–0.90)	0.038		
IV	0.333 (0.03–3.20)	0.341		
Smoking, n (%)	0.531 (0.20–1.35)	0.187		
Previous biliary intervention, n (%)	1.864 (0.79–4.35)	0.150		
WBC, mean ± SD	0.933 (0.80–1.08)	0.369		
Platelet ×10^3^, median (range)	0.741 (0.50–1.08)	0.125		
Hemoglobin in g/dL, median (range)	0.997 (0.85–1.15)	0.970		
BUN in mg/dL, median (range)	1.001 (0.94–1.06)	0.967		
Creatinine in mg/dL, median (range)	0.734 (0.25–2.07)	0.561		
Total bilirubin in mg/d/L, median (range)	0.981 (0.92–1.04)	0.538		
Albumin in g/L, mean ± SD	0.951 (0.89–1.01)	0.128		
Preoperative cholangitis, n(%)	12.193 (1.07–138.54)	0.044	10.722 (2.31–49.75)	0.002
Operative time in min, median (range)	1.126 (0.83–1.51)	0.428		
Blood loss in ml, median (range)	1.264 (1.01–1.59)	0.046		
Complications, n (%)				
Pancreatic fistula				
No	1		1	
Yes	8.399 (3.10–22.71)	0.000	6.531 (2.30–18.51)	0.000
Bile leakage	2.482 (0.73–8.44)	0.145		
Delayed gastric emptying	1.733 (0.53–5.63)	0.360		
Chyle leakage	1.978 (0.38–10.24)	0.416		
Diagnosis, n (%)				
Benign	1			
Malignant	0.952 (0.41–2.18)	0.908		

*SSI* Surgical site infection, *OR* Odds ratio, *CI* Confidence interval, *BMI* Body mass index, *ASA* American Society of Anesthesiologists, *WBC* White blood cell count, *BUN* Blood urea nitrogen

### Bacteriology

In the SSI group, the bacterial cultures were positive in 46 of 90 patients, with polymicrobial culture results in 22 patients (48%). *Enterococcus* was the most common species identified, followed by *Escherichia coli* and *Klebsiella pneumoniae*. The results of the bacterial cultures from SSI are shown in Table 4. The results of the bile cultures from the patients who experienced preoperative cholangitis are shown in Table 5. *E. coli, Enterococcus*, and *Klebsiella pneumoniae* were the common pathogens found to contaminate the bile juice.

**Table 4:** Pathogens isolated from bile juice of patients who developed preoperative cholangitis

Bacterial species	Number of cases in which each species was identified
*Enterococcus*	5
*Escherichia coli*	8
*Klebsiella pneumoniae*	5
*E. coli* ESBL	1
*Klebsiella* ESBL	2
*Pseudomonas*	1
Others	5

*ESBL* Extended spectrum beta-lactamase-producing bacteria

**Table 5:** Pathogens isolated from infected sites of surgical site infection patients

Bacterial species	Number of cases in which each species was identified
*Enterococcus*	18
*Escherichia coli*	10
*Klebsiella pneumoniae*	9
*E. coli* ESBL	6
*Klebsiella* ESBL	5
*Pseudomonas*	2
*Acinetobacter*	2
Others	22

*ESBL* Extended spectrum beta-lactamase-producing bacteria

## Discussion

The postoperative mortality rate of PD has been decreasing for many years. However, the morbidity rate remains high, particularly regarding SSI, which is one of the most common problems after PD. The incidence of SSI after PD varies from 12 to 51% [7, 8, 15–17]. Many factors contribute to SSI, including multiple enteric anastomoses, extensive physiologic changes, blood loss, and a long operative time [17]. SSI results in a large burden on the healthcare system. One study found that SSI resulted in an additional 12 days of hospitalization and approximately 5000 USD in direct costs [18], while another showed that SSI resulted in a two-fold increase in the duration of hospitalization and a 2.5-fold increase in healthcare expenditures [19]. The present study found similar results; the incidence of SSI was 32%, and the length of hospital stay in the SSI group was 28 days, which was twice as long as that in the non-SSI group.

POPF is a common and concerning problem after PD. [20, 21] Clinically relevant POPF after PD reportedly occurs in 12% of patients and is associated with a mortality rate of 39% [22]. The autodigestive effect of leaky pancreatic juice causes circumferential tissue damage and promotes bacterial infection [23]. Therefore, POPF has been reported as an independent risk factor for SSI; the incidence of serious infection in patients with POPF is reportedly 61%, which is three times higher than that in patients without POPF [9]. The present study demonstrated similar results, with POPF identified as a risk factor for SSI. Nearly half of the patients with POPF developed SSI, whereas only 17% of patients without POPF developed SSI; the incidence of SSI in patients with POPF was three times higher than that in patients without POPF. Focusing on the depth of SSI, POPF was a risk factor for both incisional SSI (OR 2.78) and organ/space SSI (OR 6.531). In the organ/space SSI group, the incidence was five times higher than that in patients without POPF. A previous study demonstrated a significant correlation between POPF and organ/space SSI, which developed in 76% of patients with POPF. The authors postulated that once POPF occurs, intra-abdominal infection may be inevitable; therefore, effective drainage and intensive antibacterial treatment should be performed to prevent further morbidity [8].

Obstructive jaundice is a common presentation of periampullary cancer. In the past, biliary drainage was routinely performed before PD, and some studies suggested that this method improved surgical outcomes [24, 25]. .However, the wisdom of routine preoperative biliary drainage has been questioned recently. A recent meta-analysis demonstrated that preoperative biliary drainage does not have a beneficial effect on postoperative outcomes and actually increases overall complications and the incidence of wound infections [26]. Two other studies demonstrated that biliary stent insertion induces bile infection, with the bile infection rate in stented patients is nearly twice that in non-stented patients (30% vs 18 and 78% vs 36%, respectively) [27, 28]. Another study revealed that bile infection is significantly associated with overall morbidity, infection morbidity, and mortality [29, 30]. Furthermore, isolated bacteria in bile are strongly associated with infectious complications (69–100%), which suggests that postoperative infection results from intraoperative contamination by biliary organisms [27, 31]. The present study showed that preoperative cholangitis, which is the result of severe bacterial infection in the biliary system, was an independent risk factor for organ/space SSI. It increased the risk for developing organ/space SSI by 10 times compared to non-preoperative cholangitis patients. Additionally, the present study demonstrated that preoperative biliary drainage was a risk factor for overall SSI, as 81% of patients with SSI had undergone preoperative biliary drainage. However, when focusing on the depth of SSI, the preoperative biliary intervention was the independent risk factor only for incisional SSI and not for organ/space SSI. The result seemed extraordinary, but the data in the literature showed a debatable result. Some studies have reported that preoperative biliary drainage is associated with a high incidence of intraabdominal abscess formation after PD [32, 33], but some studies have reported contradictory results. A study from Johns Hopkins reported no difference in the incidence of intraabdominal abscess in the preoperative drainage group compared to the non-drainage group (4% vs 6%) [34]. Another study from the M.D. Anderson Cancer Center reported that the rate of intraabdominal abscess after PD was comparable in preoperative drainage and non-drainage groups (7% vs 11%) [35]. Two other studies reported that preoperative biliary drainage was an independent risk factor for incisional SSI but was not a risk factor for organ/space SSI in hepatobiliary and pancreatic surgery [16, 36]. Nonetheless, according to recently published data, preoperative biliary drainage appears to increase the morbidity rate in patients undergoing PD, particularly in terms of septic complications, and routine biliary drainage should be avoided. A recent literature review suggests that the indications for preoperative biliary drainage are 1) acute cholangitis, obstruction with a high bilirubin level, and severe pruritus; 2) anorexia with a poor nutritional status; 3) jaundice associated with renal failure or other comorbidities; and 4) PD delayed by more than 4 weeks [37].

In SSI patients, approximately half of the bacterial cultures were positive, and 48% of these positive cultures were polymicrobial. The bacteria that commonly caused SSI were gut-derived species, such as *Enterococcus*, *Escherichia coli* and *Klebsiella pneumoniae*, which were the common pathogens causing preoperative cholangitis. Extended-spectrum beta-lactamase-producing bacteria were identified in 24% of positive cultures in SSI patients and were identified in some preoperative cholangitis patients. Thus, when preoperative cholangitis or SSI is suspected, antibiotics targeting these kinds of bacteria should be considered until the responsible bacteria are otherwise identified.

The incidence of DGE was significantly higher in the SSI group than in the non-SSI group. Septic complications, especially intraabdominal collection, might affect the function of the alimentary tract. Appropriate management, including appropriate antibiotics and abdominal collection drainage, is mandatory to improve clinical outcomes and reduce further morbidities. A long hospital stay is an inevitable consequence in patients who experience SSI because of the need for further treatments. In the SSI group, the duration of drain placement was longer than that in the non-SSI group. This might be because of the higher incidence of POPF in those patients. In our practice, the drains were removed only when the pancreatic fistula had completely disappeared.

Various risk factors for SSI have been reported in recent studies, which are shown in Table 6. Many studies have demonstrated that preoperative biliary drainage [2, 7, 8, 16, 17, 36, 38] and pancreatic fistula [8, 10, 38] are independent risk factors for SSI, which is consistent with the present study. Previous studies [8, 30, 36] have reported that a high BMI is a risk factor for SSI, which is consistent with another study in the literature showing that overweight and obesity increase the risk of SSI in many types of surgery [39]. The present study showed that the BMI in the SSI group was significantly higher than in the non-SSI group; however, it was not a significant risk factor for SSI in multivariate analysis. This might be because of the small number of patients included in the study. Two other previous studies [8, 30] also demonstrated that a long operative time (> 480 min, > 7 h) was a risk factor for developing SSI. The data of the present study did not reveal the same result. The difference in practice, for instance, the type and frequency of prophylactic antibiotic redosing, saline irrigation before abdominal closure and a different number of patients included in the studies, might have influenced the different results. Blood transfusion [9, 30] and neoadjuvant therapy [2, 38] have also been described as risk factors for SSI. The present study did not have sufficient data to discuss these factors. However, blood transfusion is still a debatable topic because studies in the literature have shown contradictory results [40, 41]. Neoadjuvant therapy has recently become an important modality of treatment in pancreatic cancer. To my knowledge, there have been few publications describing this issue, so more studies are needed to draw conclusions.

**Table 6:** Summarization of recent studies of risks of surgical site infection after pancreaticoduodenectomy

Author (year)	Type of study	No. of patients	Incidence of SSI (%)	Percent of malignancy (%)	Risk factors of surgical site infection
Poluk K. E (2016) [37]	Single center retrospective	679	17.2	–	Pre-operative biliary drainage, Neoadjuvant chemotherapy
Zhang L (2016) [9]	Single center retrospective	212	29^a^	80	Pancreatic fistula, Blood transfusion
Barreto S. G (2015) [7]	Single center retrospective	277	35	81	Pre-operative biliary stent insertion, Non-diabetic endocrine co-morbidity.
De Pastena M (2017) [17]	Single center retrospective	893	45.8†	–	Pre-operative biliary drainage
Sugiura T (2012) [8]	Single center retrospective	408	51	90	Long operative time, High Body Mass Index, Pancreatic fistula, Semi-close suction drain, Main pancreatic duct < 3 mm, Abdominal wall fat thickness > 10 mm.
Burkhart R. A (2017) [2]	Single center retrospective	394	19.8	71.1	Pre-operative biliary drainage, neoadjuvant therapy, prior abdominal surgery
Shinkawa H (2018) [10]	Single center retrospective	106	14.2	–	Nutritional risk index, Pancreatic fistula
Gavazzi F (2016) [35]	Single center retrospective	180	52.3	86	Pre-operative biliary stent, Cardiac disease, BMI > 25 mg/m^2^
Okano K (2015) [30]	Multi center retrospective	4147	27.1	90	Male sex, age 70 years or more, Body mass index at least 25 kg/m^2^, Other previous malignancy, Liver disease, Bile contamination, Duration of surgery 7 h or longer, Intraoperative blood transfusion, Soft pancreas

^a^ Overall infectious complication

- Not stated

The present study had two main limitations. First, because of its retrospective nature, some selection bias may have been present. Second, the study population was relatively small compared with previous studies.

## Conclusions

Preoperative biliary drainage, POPF, and preoperative cholangitis are significant risk factors for SSI. To lower the incidence of SSI, preoperative biliary drainage should be performed only in select cases, and every effort should be made to reduce the incidence of POPF and preoperative cholangitis.

## Data Availability

The clinical datasets supporting the results of this article are available from the corresponding author.

## Abbreviations

SSI: Surgical site infection
PD: Pancreaticoduodenectomy
BMI: Body mass index
POPF: Postoperative pancreatic fistula
OR: Odds ratio
CI: Confidence intervals
